# Charge storage mechanisms of manganese oxide nanosheets and N-doped reduced graphene oxide aerogel for high-performance asymmetric supercapacitors

**DOI:** 10.1038/srep37560

**Published:** 2016-11-18

**Authors:** Pawin Iamprasertkun, Atiweena Krittayavathananon, Anusorn Seubsai, Narong Chanlek, Pinit Kidkhunthod, Winyoo Sangthong, Santi Maensiri, Rattikorn Yimnirun, Sukanya Nilmoung, Panvika Pannopard, Somlak Ittisanronnachai, Kanokwan Kongpatpanich, Jumras Limtrakul, Montree Sawangphruk

**Affiliations:** 1 Department of Chemical and Biomolecular Engineering, School of Energy Science and Engineering, Vidyasirimedhi Institute of Science and Technology, Rayong 21210, Thailand; 2 Department of Chemical Engineering, Kasetsart University, Bangkok 10900, Thailand; 3 Synchrotron Light Research Institute (Public Organization), 111 University Avenue, Muang District, Nakhon Ratchasima 30000, Thailand; 4 Department of Chemistry, Kasetsart University, Bangkok 10900, Thailand; 5 School of Physics, Institute of Science, Suranaree University of Technology, Nakhon Ratchasima 30000, Thailand; 6 Department of Applied Physics, Faculty of Sciences and Liberal Arts, Rajamangala University of Technology Isan, Nakhon Ratchasima 30000, Thailand; 7 Frontier Research Centre (FRC), Vidyasirimedhi Institute of Science and Technology, Rayong 21210, Thailand; 8 Department of Materials Science and Engineering, Vidyasirimedhi Institute of Science and Technology, Rayong 21210, Thailand

## Abstract

Although manganese oxide- and graphene-based supercapacitors have been widely studied, their charge storage mechanisms are not yet fully investigated. In this work, we have studied the charge storage mechanisms of K-birnassite MnO_2_ nanosheets and N-doped reduced graphene oxide aerogel (N-rGO_ae_) using an *in situ* X-ray absorption spectroscopy (XAS) and an electrochemical quart crystal microbalance (EQCM). The oxidation number of Mn at the MnO_2_ electrode is +3.01 at 0 V vs. SCE for the charging process and gets oxidized to +3.12 at +0.8 V vs. SCE and then reduced back to +3.01 at 0 V vs. SCE for the discharging process. The mass change of solvated ions, inserted to the layers of MnO_2_ during the charging process is 7.4 μg cm^−2^. Whilst, the mass change of the solvated ions at the N-rGO_ae_ electrode is 8.4 μg cm^−2^. An asymmetric supercapacitor of MnO_2_//N-rGO_ae_ (CR2016) provides a maximum specific capacitance of ca. 467 F g^−1^ at 1 A g^−1^, a maximum specific power of 39 kW kg^−1^ and a specific energy of 40 Wh kg^−1^ with a wide working potential of 1.6 V and 93.2% capacity retention after 7,500 cycles. The MnO_2_//N-rGO_ae_ supercapacitor may be practically used in high power and energy applications.

Supercapacitors or electrochemical capacitors are energy-storage devices widely used in many high-power applications^1^,^2^. They have high specific power (~10 kW kg^−1^) and long cycle life (up to 500,000 cycles)^3^ when compared with batteries^4^. This is because the charge storage mechanisms of supercapacitors are mainly at the solid-liquid interface via electrochemical double layer capacitive (EDLC) and pseudocapacitive behaviors. On the other hand, the batteries store charges via redox reactions based on intercalation chemistry^5^. Improvement in the specific energy of the supercapacitors while keeping their high specific power and capacitance retention is therefore a focal point in the supercapacitor research area.

Supercapacitors are classified to be either symmetric or asymmetric depending on the materials used at the positive and negative electrodes. The difficulty in developing symmetric supercapacitor, which use the identical material at both positive and negative electrodes, is that a single material will only prefer either solvated positive or negative ions. The charge storage performance of the symmetric supercapacitor is therefore limited by the electrode where it can store less charge. To solve this problem, asymmetric supercapacitors (ASCs) using different materials at the positive and negative electrodes are of interest since they can provide higher charge storage performance with wider working potentials^6^. The maximum charge storage capacity of the ASCs can be finely tuned and achieved by using proper materials and compositions at positive and negative electrodes. Therefore, the recent effort has been devoted to developing the electrode materials of the advanced ASCs. Recently, the ASC of the polypyrrole nanotubes (positive electrode)//N-doped carbon nanotubes (negative electrode) can provide a wide working potential of 1.4 V, a specific energy of 28.95 Wh kg^−1^ with a specific power of 7.75 kW kg^−1^ and a cyclic stability of ca. 90% retention after 2,000 cycles^7^. The ASC of Ni–Co hydroxide@reduced graphene oxide//3D porous carbon exhibits a specific energy of 56.1 Wh kg^–1^ with 80% retention after 17,000 cycles^8^. Note, Ni and Co hydroxides are battery-like electrode materials. The ASC of the MnO_2_ nanosheet//carbon fibers displays a specific capacitance of 87.1 F g^−1^ and a specific energy of 27.2 Wh kg^−1^ with 95% capacitance retention over 3,000 cycles^9^. The ASC of Fe_2_N //TiN exhibits 5.4 Wh kg^−1^ and specific power of ca. 6.4 kW kg^−1^ with 98% capacity retention in 20,000 cycles^10^. The ASC based on Ti-doped Fe_2_O_3_@PEDOT//MnO_2_ provides an energy density of 0.89 mWh cm^−3^ with about 85% retention capacitance after 6,000 cycles^11^. To further improve the charge storage performance of the ASCs, the positive and negative electrode materials with high ionic and electronic conductivities, porosity, and surface area are needed. In this work, new advanced ASCs have been fabricated using MnO_2_ nanosheets and nitrogen-doped reduced graphene oxide aerogel (N-rGO_ae_) as positive and negative electrodes, respectively.

Among transition metal oxide materials, MnO_2_ is well recognized as a good candidate for the positive electrode due to its wide potential range in the positive side and high theoretical specific capacitance, high stability, low cost, abundance, and no environmental hazard^12^,^13^,^14^,^15^. Lee and Goodenough firstly presented that the amorphous MnO_2_.H_2_O used as an active material for the supercapacitors in KCl solution exhibited a specific capacitance of ca. 200 F g^−1^ 16. Toupin, M. *et al*. reported a change in the oxidation state between Mn^3+^ and Mn^4+^ during the charge/discharge process of the MnO_2_ electrode using an *ex situ* X-ray photoelectron spectroscopy (XPS) measurement of the dried MnO_2_ electrode after polarized^17^. They also reported that the charge compensation of the Mn^3+^ a reduced state is due to Na^+^ and H^+^ adsorption^17^. In contrast, Xu, C. *et al*. studied the charge storage mechanism of the MnO_2_ by controlling the pH of the electrolytes and reported that the cations of the electrolyte rather than H^+^ are responsible for the pseudocapacitance of MnO_2_^18^. *In operando* Raman spectroscopy was also employed to probe the structural changes of the α-MnO_2_ electrode during the charge/discharge process for which the charge storage mechanism is based on the intercalation chemistry^19^. As the results, it can be concluded here that the charge storage mechanisms of MnO_2_-based supercapacitors are not yet fully clear.

Interestingly, the mixed valent MnO_x_ including MnO_2_ and Mn_3_O_4_ recently reported exhibits superior charge storage performance than individual MnO_2_ or Mn_3_O_4_^20^,^21^,^22^. Among several methods for the preparation of MnO_x_ nanostructures on conductive substrates including precipitation^23^, sol-gel^16^, and electrodeposition^24^, the electrodeposition is well-recognized as an efficiency method with high homogeneity active species. It is also simple, scalable, and cheap technique^25^. Also, this technique does not require the polymer binders (e.g., PVDF, PTFE), which can introduce many disadvantages including an obstacle for the movement of ions and electronic charge transport. It is also necessary to note here that the charge storage mechanisms of the mixed valent MnO_x_ have not yet been investigated. Thus, understating how the Mn oxidation states do change during the charging/discharging processes is crucial to the development of this material.

For the negative electrodes, the N-rGO_ae_ with high surface area and porosity, which are good for supercapacitor electrodes. The diluted N- and O-containing groups of the N-rGO_ae_ can lead to high ionic adsorption^26^. They can also store the electronic charges via surface redox reactions^27^,^28^. Interconnected 3D graphene structure can enhance the diffusion of solvated ions via a capillary force providing ultrahigh specific powder^2^. However, an important question how much solvated ionic charges can be stored by the N-rGO_ae_ has not yet been reported. Electrochemical quartz crystal microbalance (EQCM) is then used in this work to address this issue during the charging/discharging processes.

In this work, MnO_2_ nanosheets with a birnessite structure having negatively charged MnO_2_ layers along with K^+^ counter ions and water among the adjacent layers were synthesized by a potential-step electrodeposition. The oxidation number of Mn in MnO_2_ nanosheets during the charging/discharging processes was subsequently monitored by an *in situ* X-ray absorption spectroscopy (XAS). In addition, the mass change on the electrodes of MnO_2_ and N-rGO_ae_ during charging/discharging was evaluated by an *in situ* EQCM. The results provide further understanding on the charge storage mechanisms of MnO_2_ nanosheets and N-rGO_ae_.

## Results and Discussion

### Morphologies of as-synthesised materials

**Figure 1:**
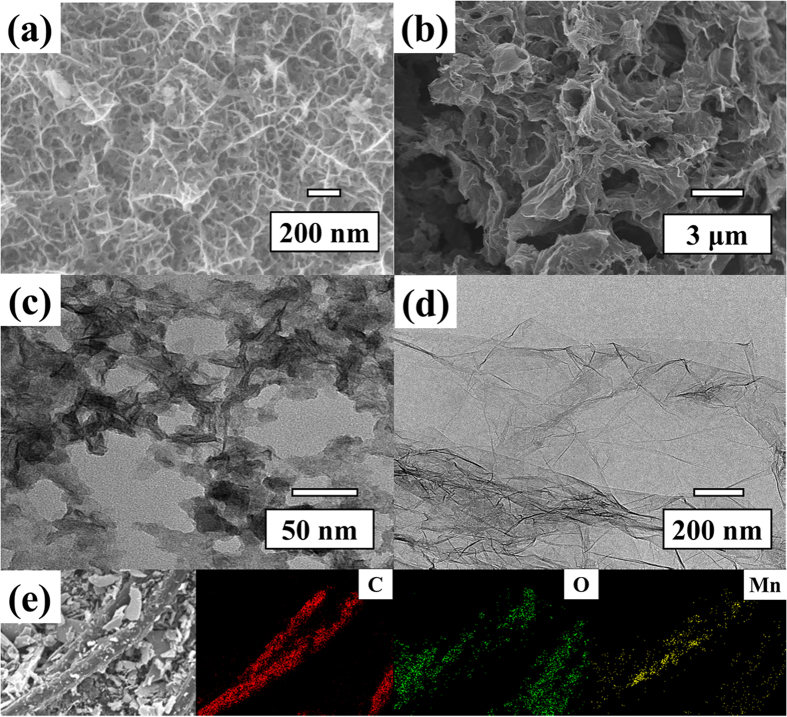
FE-SEM images of (**a**) MnO_2_ and (**b**) N-rGO_ae_ as well as TEM images of (**c**) MnO_2_ and (**d**) N-rGO_ae_ and (**e**) EDX mapping of MnO_2_/c-CFP mainly containing C, O, and Mn elements.

The morphology of the MnO_2_ synthesized using the potential-step electrodeposition was characterized by FE-SEM as shown in Fig. 1a. The as-electrodeposited MnO_2_ is rather porous with a pore diameter of ca. 10–50 nm due to the interconnection of the MnO_2_ nanosheets. This morphology is ideal for the supercapacitor electrode since it can enhance the mass transport of the electrolyte due to the capillary force^29^,^30^. Figure 1b shows an FE-SEM image of N-rGO_ae_ illustrating a few layers of overlapping graphene sheets forming the framework structure with a pore diameter of 0.2–3 μm. The N-rGO_ae_ exhibits ultrahigh porosity, which can also accelerate the electrolyte diffusion on the negative electrode. Figure 1c displays a TEM image of the MnO_2_ for which the morphology of the as-electrodeposited MnO_2_ is a sheet-like shape with a diameter of ca. 20–50 nm. The MnO_2_ nanosheets connect to each other forming a porous structure. Figure 1d shows a TEM image of N-rGO_ae_, which is nearly transparent containing many wrinkles of the N-rGO_ae_ framework structure. In addition, the EDX mapping of the MnO_2_ coated on the c-CFP substrate in Fig. 1e displays three main elements, which are C, O, and Mn with 40.2, 27.0, and 18.7% by atomic weight, respectively. The 14.1% remaining element is F, which comes from the carboxyl-modified carbon fiber paper (c-CFP) substrate since a PTFE is used as a binder in the production process of the CFP^13^.

### Structures of as-synthesised materials

**Figure 2:**
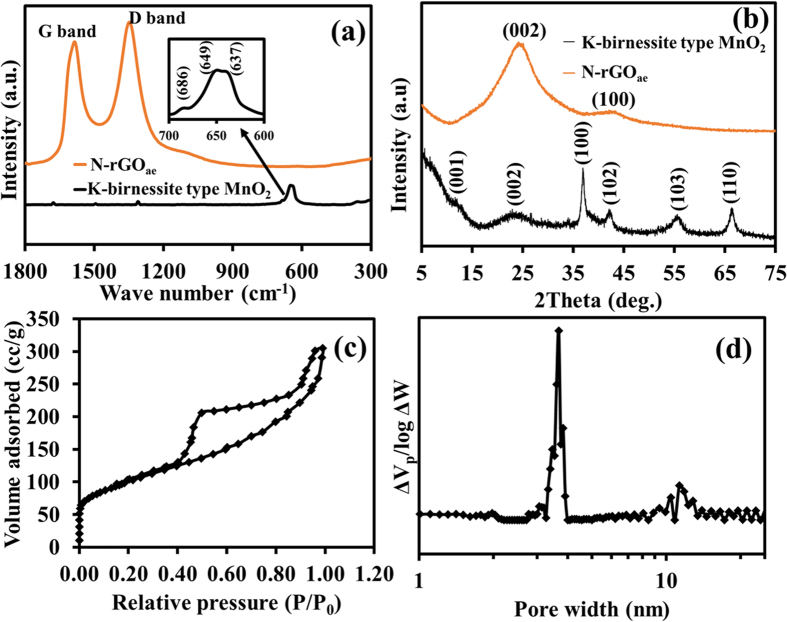
(**a**) Raman spectra, (**b**) XRD patterns of MnO_2_ and N-rGO_ae_ and (**c**) N_2_ sorption isoterm and pore size distribution of N-rGO_ae_.

To further study the physical and chemical properties of the as-synthesized materials, Raman, XRD, and XPS techniques were carried out. In Fig. 2a, Raman spectra of the MnO_2_ display two main contribution peaks of the MnO_2_. N-rGO_ae_ displays two distinct peaks at 1,350 and 1,580 cm^−1^ according to the normal characteristic peaks of the rGO materials. Generally, the D-band at 1,350 cm^−1^ represents the amount of the disordered carbon structure, which consists of the sp^3^ carbon atoms at the edge of graphitic sheets. The G-band at 1,580 cm^−1^ illustrates the vibrational mode of the graphitic sp^2^carbon sheets^31^. Additionally, the defect ratio (I_D_/I_G_) of N-rGO_ae_ is 1.05, which is in good agreement with other previous work^32^. The amorphous carbon content of N-rGO_ae_ calculated from the deconvoluted peaks at around 1,510 cm^−1^ is ca. 17.7%.

The XRD pattern of N-rGO_ae_ (Fig. 2b) displays two broad peaks at 2θ of 24.3 and 43.7° referring to the characteristics of rGO^33^. The XRD pattern of the as-electrodeposited MnO_2_ indicates the K-birnessite MnO_2_ nanosheets (JCPDS 80-1098)^34^,^35^. The peaks at 2θ about 12, 24, 37, 43, 56, and 66° are due to (0 0 1), (002), (111), (−112), (113), (020), and (220) planes showing a lamellar structure. The structure consists of single sheets of edge-sharing [MnO_6_] octahedral and water molecules and K^+^ between the adjacent layers^34^,^35^,^36^. The orthogonal distance between two consecutive slabs of [MnO_6_] is ca. 7.3 Å. The mixed vacancy of manganese ions in K-birnassite MnO_2_ nanosheets plays an important role of a spontaneous redox reaction enhancing the pseudocapacitance of ASCs^22^. In addition, the N_2_ gas adsorption was carried out to determine the specific surface area and pore size distribution of N-rGO_ae_ as shown in Fig. 2c and d. By following the IUPAC classification, the gas adsorption isotherm of N-rGO_ae_ is in a type-IV isotherm (a hysteresis loop type II) owing to interconnected pore networks (see Fig. 2c). The BET surface area of N-rGO_ae_ is about 352 m^2^ g^−1^ having an average pore width of 3.7 nm (Fig. 2d).

### Surface analysis of as-fabricated electrodes

**Figure 3:**
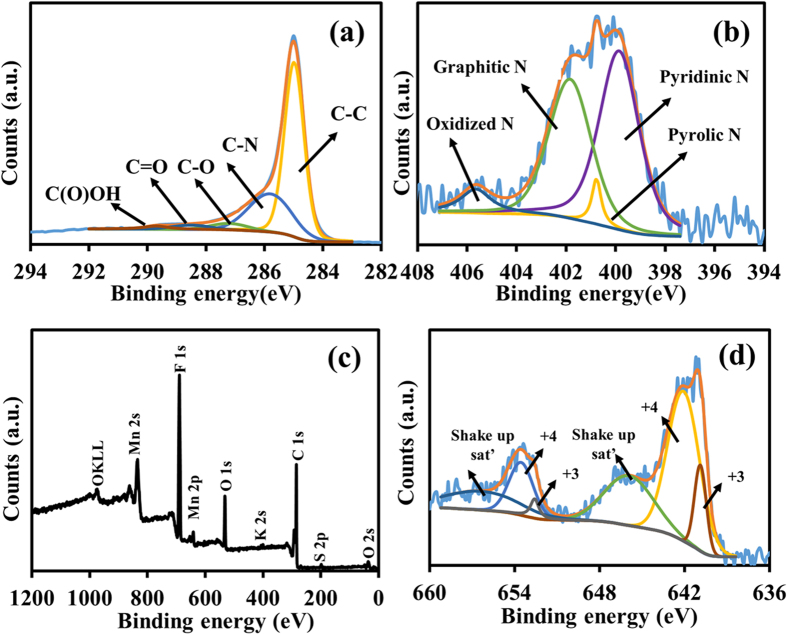
(**a**) C1s and (**b**) N1s XPS spectra of N-rGO_ae_ on c-CFP, (**c**) XPS survey, and (**d**) Mn2p XPS of MnO_2_.

The surface chemical composition of the as-fabricated electrodes was analyzed by the XPS technique. The C1s spectra of N-rGO_ae_ sprayed on c-CFP (Fig. 3a) display four main peaks at 284.9, 285.2, 286.1, and 288.5 eV corresponding to C-C, C-N, C-O and C=O, respectively^37^. The diluted nitrogen content in Fig. 3b can be deconvoluted into four peaks of pyridinic N (399.8 eV), pyrolic N (400.5 eV), graphitic N (401.7 eV) and oxidized N (405.4 eV), respectively^38^. The graphitic N can improve the charge transfer in the rGO matrix^39^. Other N-functional groups (Pyrolic N and Pyridinic N) also play an important role for the pseudocapacitance^27^,^28^,^40^.

A wide-scan XPS spectrum of MnO_2_ coated on c-CFP is shown in Fig. 3c confirming the elements of the as-prepared electrode. Notably, K is also found on the XPS spectrum since K^+^ is a balance charge of negatively-charged MnO_2_ layers. The Mn2p spectra of the as-electrodeposited MnO_2_ coated on c-CFP in Fig. 3d show two broad peaks of Mn2p_3/2_ and Mn2p_1/2_, which can be deconvoluted to many peaks corresponding to two different oxidation states of Mn species^41^. This material has the advantage of mixed valent MnO_2_ for supercapacitors. Each broad peak can be classified into three parts at 640.9, 642.1, and 645.9 eV for Mn2p_3/2_ and 652.6, 653.8, and 656.2 eV for Mn2p_1/2_. The peaks at 640.9 and 652.6 eV are the characteristics of Mn^3+^ (21.3%) while those at 642.1 and 653.8 eV are the characteristics of Mn^4+^ (78.7%) as well as those at 645.9 and 656.2 eV are attributed to shakeup satellites. For the O1s XPS spectrum, which is not shown here, the spectrum contains three main peaks located at 531.4, 532.6, and 533.1 eV attributing to Mn-O-H, H-O-H and O-C, respectively^42^.

### Electrochemical evaluatioon of as-fabricated electrodes

**Figure 4:**
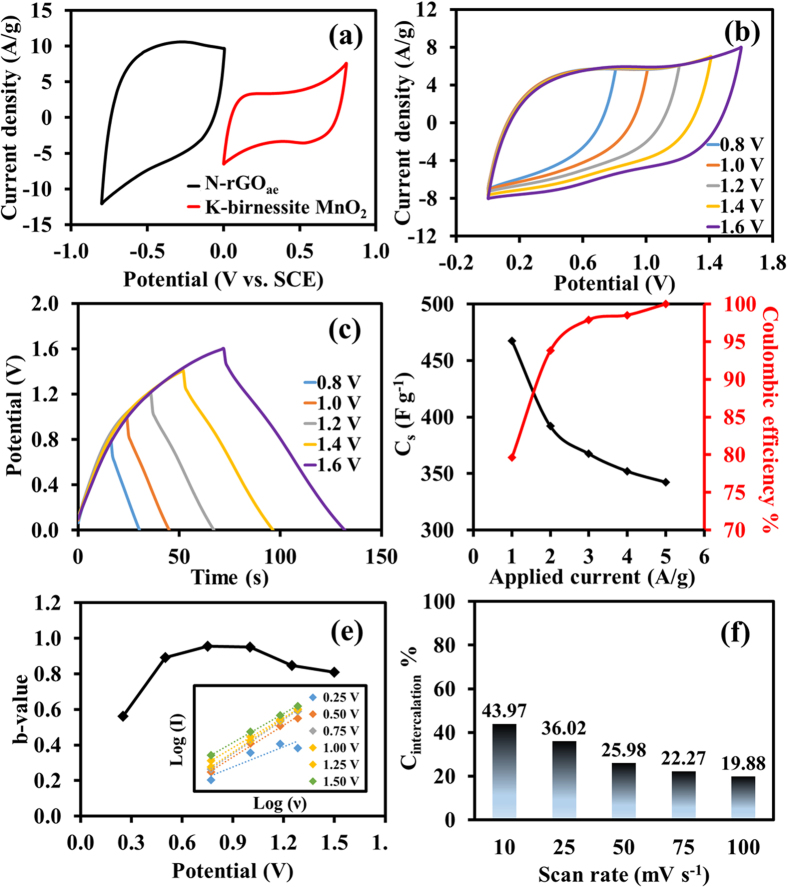
(**a**) CVs of the as-fabricated electrodes at 25 mV s^−1^ and (**b**) CVs at different working potentials (50 mV s^−1^), (**c**) GCDs at different working potentials (5 A g^−1^), and (**d**) specific capacitance and coulombic efficiency vs. applied current density, (**e**) the *b* value as a function of potential, and (**f**) the bar chart of the diffusion-controlled intercalation capacitance vs. scan rates of as-fabricated MnO_2_//N-rGO_ae_ supercapacitor devices.

To evaluate the electrochemical property of the as-fabricated electrodes, a three-electrode system using the as-synthesized material as a working electrode, a Pt wire as a counter electrode, and a saturated calomel electrode (SCE) as a reference electrode was carried out in 0.5 M Na_2_SO_4_ solution. The optimized potential range of the as-prepared electrodes is shown in CVs (Fig. 4a). Also, an optimum mass ratio finely tuned between the mass of active materials at positive and negative electrodes (m^+^/m^−^) is 1.75 providing the highest charge storage performance calculated by a charge balance according to Eq. 1^43^ as follow;


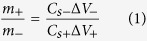


where *m* is the mass of the active material, *C*_*s*_ is the specific capacitance, and *ΔV* is the voltage range for positive and negative electrodes^44^. Note, all electrochemical properties of half-cell MnO_2_ and N-rGO_ae_ electrodes are shown in Figure S1 and 2 of the supporting information, respectively. The MnO_2_//N-rGO_ae_ supercapacitor was then assembled using a hydrolyzed PE containing 0.5 M Na_2_SO_4_ as a separator. The operating potential window was varied from 0.8–1.6 V as shown in Fig. 4b. The rectangular-shaped CVs with a broad redox peak indicate the pseudocapacitive behavior that comes from the surface redox reaction of MnO_2_^15^. Surprisingly, the as-fabricated device levels off at the same range of the current density until 1.6 V. Moreover, GCDs show a symmetrical shape relating to the CV result (Fig. 4c). The MnO_2_//N-rGO_ae_ device exhibits the specific capacitances of 467.38–342.43 F g^−1^ (4.55–3.33F cm^−3^) at 1.0–5.0A g^−1^, respectively. The device has high coulombic efficiency up to 100% at 5 A g^−1^ (Fig. 4d). To further study the capacitive effect, *b* value was calculated from a power law according to Eq. 2^45^ as follow;


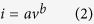


where *i* is the current and *ν* is the scan rate. Both *a* and *b* are the constant parameters for which the *b* value can be determined from a slope of the linear plot between log *i* vs. log *ν (see inset graphs in* Fig. 4e and Figure S3a). According to power law relationship, *i* is equal to *aν* for non-diffusion limited processes and *i* is equal to *aν*^*1/2*^ for diffusion limited processes. Typically, the *b* value is equal to 1.0 for non-diffusion-controlled surface capacitive and equal to 0.5 for diffusion-controlled redox reaction, which is a typical battery behavior^44^. The result in Fig. 4e shows that the *b* values in this work are 0.56, 0.89, 0.95, 0.95, 0.84, 0.80 at the potentials of 0.25, 0.50, 0.75, 1.00, 1.25, and 1.50 V, respectively. This can confirm that the devices have both EDLC and surface redox reactions. In addition, the percentage of intercalation and capacitive contribution calculated by Eq. 3^46^ is shown in Fig. 4f. The intercalation capacitance decreases when increasing scan rates due to the diffusion limit of the electrolytes.


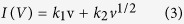


where *k*_*1*_ and *k*_*2*_ are the slope and interception, respectively, which can be determined from Figure S3b.

**Figure 5:**
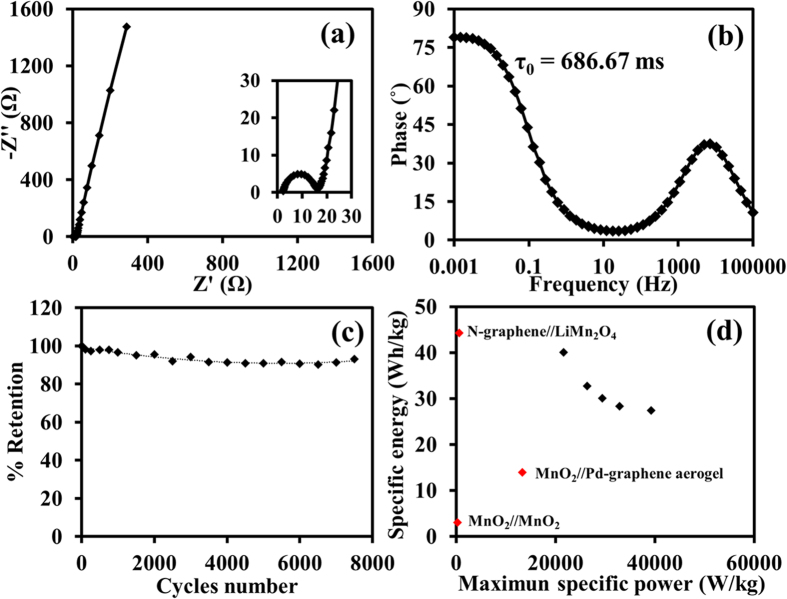
(**a**) Nyquist plot, (**b**) phase vs. frequency, (**c**) capacitance retention over 7,500 cycles, and (**d**) Ragone plots of the MnO_2_//N-rGO_ae_ supercapacitor compared with other previous work^49^,^50^,^51^.

Besides, the EIS result of the MnO_2_//N-rGO_ae_ supercapacitor at a sinusoidal signal of 10 mV from 100 kHz to 1 mHz is shown in the Nyquist plots (Fig. 5a). The straight line of the Nyquist plots increases sharply at a low frequency region to the Y-axis indicating almost the ideal supercapacitor dominated by the capacitive behavior from the formation of ionic and electronic charges. At high frequency, the electronic charge transfer resistance (*R*_*ct*_) due to the surface redox of MnO_2_ is about 13.45 Ω with an internal resistance (*R*_*s*_) of 2.32 Ω located at the interception on the X-axis. In addition, the relaxation-time constant (τ_0_), which is a minimum time required to discharge for all stored charges, can be determined from an inversion of the frequency at the maximum phase angle as shown in Fig. 5b. It is necessary to note the smaller value of τ_0_ the higher power of the supercapacitors^47^. In this work, τ_0_ is about 686 ms, which is much smaller than other previous report^1^,^48^. Finally, the stability of the MnO_2_//N-rGO_ae_ supercapacitor evaluated by the GCD method over 7,500 cycles at 5 A g^−1^ (Fig. 5c) is over 93.2% retention. The as-assembled device provides the highest specific energy of 40 Wh kg^−1^ and the highest specific power of 39 kW kg^−1^, which are much higher than those of other previous related report (see Fig. 5d)^49^,^50^,^51^. Note, CVs at different scan rates, GVDs at different specific currents, and the calculated specific capacitances at different frequencies of the device are also shown in Figure S4 of the supporting information.

### *In situ* X-ray absorption spectroscopy

**Figure 6:**
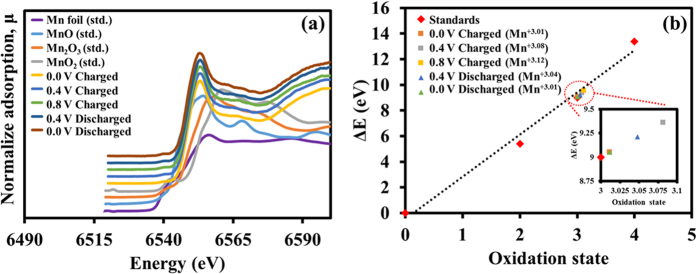
(**a***) In situ* high-resolution Mn K-edge fluorescence XAS spectra of the as-prepared MnO_2_ electrodes and Mn standard compounds and (**b**) the oxidation states vs. ΔE (eV) of the MnO_2_ electrodes during charging/discharging by a chronoamperometry method at applied potentials from 0.0–0.8 V vs. SCE and backward potentials. Note, the XAS was carried out after reaching the steady state.

In order to clarify the origin of the remarkable specific energy and specific power of the MnO_2_-based supercapacitor, the charge storage behavior occurred during the charge/discharge processes has been investigated by the *in-situ* XAS measurement. As Mn in the manganese oxide with different oxidation states plays a prominent role for the surface redox reaction during the charge/discharge process, *in situ* monitoring the oxidation number change of the Mn during charge/discharge processes is therefore crucial for understanding the pseudocapacitive behavior. In this work, the *in situ* XAS technique was carried out together with the chronoamperometry in 0.5 M Na_2_SO_4_ electrolyte during applying the potentials stepped from 0.0, 0.4, and 0.8 V vs. SCE and the backward potentials from 0.8 to 0.4 V vs. SCE and from 0.4 to 0.0 V vs. SCE. Note, in order to reach the steady state, each step potential was hold for 15 min before starting the XAS measurement^52^,^53^. The Mn K-edge fluorescence energy of the MnO_2_ charged at 0.0 V vs. SCE is 6548.05 eV and the energy value increases up to 6548.36 and 6548.53 eV when the potentials were applied to 0.4 and 0.8 V vs. SCE, respectively (see Fig. 6a). The Mn oxidation states of the MnO_2_ electrode being charged at 0.0, 0.4, and 0.8 V vs. SCE are +3.01, +3.08, and +3.12, respectively (see Fig. 6b). Note, the oxidation number of the as-electrodeposited MnO_2_ is +3.79.

The XAS result here confirms the reversible redox reaction of the MnO_2_ and the proposed general redox reaction (4) below based on the intercalation/de-intercalation processes of Na^+^ and H^+^ is shown below^15^,^16^,^54^,^55^;


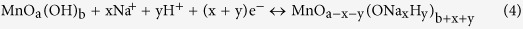


When the stepped potentials were applied backward from 0.8 to 0.4 V vs. SCE and afterward from 0.4 to 0.0 V vs. SCE, the edge energies are 6548.21 eV and 6548.05 eV, respectively (see Fig. 6a). The oxidation states of Mn in the MnO_2_ return to +3.04 at 0.4 V vs. SCE and +3.01 at 0 V vs. SCE (see Fig. 6b).

In order to clarify the charge storage mechanism of the as-prepared K-birnessite MnO_2_, the effect of the pH of 0.5 M Na_2_SO_4_ electrolyte was also studied by verying pH of the 0.5 M Na_2_SO_4_ electrolytes by adding conc. H_2_SO_4_ (see the experimental results in Figure S5 and 6 of the supporting information). It is found that at pH beween 0.08 and 1.10, the solvated H^+^ plays an important role in the charge storage capacity via an intercalation redox reaction (see redox peaks in CVs of Figure S5a) according to the reaction mechanism (5) below;


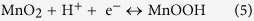


At pH 2.03–4.02, the solvated Na^+^ plays a major role to the charge storage capacity of the MnO_2_ (see the mechanism in reaction (4) above). At pH > 5.36 adjusted by adding NaOH, it was found that the specific capacitances are significantly reduced since the MnO_2_ layers having negative charge do not like to adsorb/absorb solvated anions i.e., OH^−^. As the results, we can conclude that H^+^ plays a significant role in the charge storage capacity at pH < 2.03.

### Electrochemical quartz crystal microbalance

**Figure 7:**
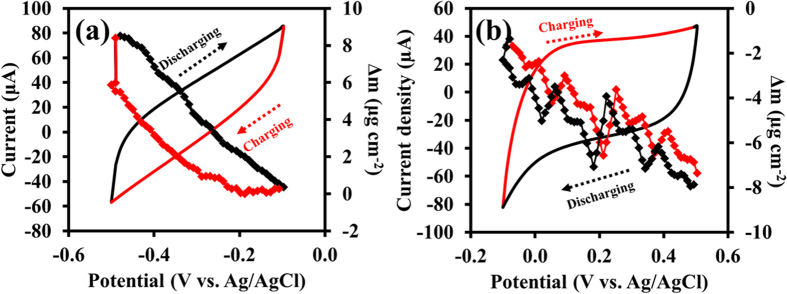
CVs at 25 mVs^−1^ and *in situ* EQCM responses of (**a**) N-rGO_ae_ and (**b**) MnO_2_ electrodes.

In addition to the *in situ* XAS results, the *in situ* gravimetric measurement of the mass changes on N-rGO_ae_ and MnO_2_ electrodes was eventually evaluated via the EQCM method. The EQCM electrode was prepared by a drop-coating of the as-prepared materials onto the Au/TiO_2_ quartz crystal surfaces. The *in situ* probing via the CV method was carried out in 0.5 M Na_2_SO_4_ solution using a three-electrode system with Ag/AgCl (3 M KCl) as the reference electrode and Au wire as the counter electrode (Fig. 7). The quartz resonance frequency (*Δf*) can be converted into the mass change (*Δm*) according to the derived Sauerbrey equation (6)^56^ below;


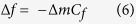


where the frequency (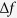) in Hz and the calibration constant (*C*_*f*_) is 0.0815 Hz ng^−1^cm^2^.

The CV and *Δm* from the quartz frequency response of N-rGO_ae_ are shown in Fig. 7a from −0.1 V to −0.5 V vs. Ag/AgCl. The CV shows a narrow potential window (about 0.6 V) because of small amount of N-rGO_ae_ coated onto the Au/TiO_2_ electrode^57^. For the charge storage mechanisms of the N-rGO_ae_ at the negative electrode, it can store ionic charges via the physical adsorption (EDLC) at the solid-liquid interface by adsorbing/absorbing the solvated ions^58^. Furthermore, the N-containing groups of the N-doped rGO can store electronic charges via the redox reaction (7) below^58^;


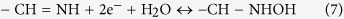


The *Δm* or mass deposited to the electrode during the charge process gradually increases to 8.4 μg cm^−2^. After discharged, the ion accumulation releases to the electrolyte and the electrode returns to the initial state. Besides, the CV of the MnO_2_ electrode in Fig. 7b displays an anodic potential range from −0.1 to 0.5 V vs. Ag/AgCl. The *Δm* is 7.4 μg cm^−2^, relating to solvated cations (i.e. Na^+^ and K^+^) inserted/released from the MnO_2_ layers. This is why the Mn oxidation state of the MnO_2_ electrode being charged is increased. After fully discharged, the *Δm* returns to the initial value again confirming the XAS result.

## Conclusions

High-performance asymmetric supercapacitor of the MnO_2_//N-rGO_ae_ has been successfully fabricated. The MnO_2_ nanosheets were prepared using a potential step electrodeposition and used as the positive electrode of the supercapacitor. The N-rGO_ae_ was synthesized using a hydrothermal process by reducing graphene oxide with hydrazine (a nitrogen source) and used as the negative electrode. The *in situ* XAS carried out together with the chronoamperometry indicates that the oxidation state of manganese ions in the MnO_2_ electrode being charged remarkably rises from +3.01 to +3.12 when applying potentials at 0 to 0.8 V vs. SCE and returns to +3.01 at 0 V vs. SCE during the discharge process. This is a reason why MnO_2_ nanosheets exhibit excellent capacity retention. The mass changes of solvated ions at the N-rGO_ae_- and MnO_2_-coated Au/TiO_2_ quartz crystal EQCM electrodes during the charge/discharge processes are ca. 8.4 and 7.4 μg cm^−2^, respectively. It is also found in this work that [H^+^] plays a significant role in the charge storage capacity at pH of the electrolyte, 0.5 M Na_2_SO_4_(aq) <2.03. At pH 2.03–4.02, the solvated Na^+^ plays a major role to the charge storage capacity of the MnO_2_. At pH > 5.36, the specific capacitance of the device is significantly reduced since the birnassite MnO_2_ layers having negative charge do not like to adsorb/absorb solvated anions i.e., OH^−^. An as-fabricated MnO_2_//N-rGO_ae_ with a finely tuned mass loading ratio of 1.75 provides a wide working potential of 1.6 V with the highest specific power and energy of 39 kW kg^−1^ and 40 Wh kg^−1^, respectively. This device with a CR2016 size has 93.2% capacity retention after 7,500 cycles at 5 A g^−1^. The enhancement in the specific energy and specific power of the MnO_2_//N-rGO_ae_ supercapacitors can compete with the batteries in many applications.

## Methods

### Preparation of flexible carboxyl-modified carbon fiber paper (c-CFP)

The c-CFP substrate was prepared by an acid treatement^13^,^29^. Briefly, conc. H_2_SO_4_ (150 ml) and conc. HNO_3_ (50 ml) were mixed together in a beaker by stirring at 100 rpm for 10 min. The CFP with 5 × 5 cm^2^ was then immersed to the acid mixture and kept stirring at 60 °C at 100 rpm for 1 h. The c-CFP was then washed with Milli-Q water 5 times and dried at 50 °C for 24 h.

### Preparation of N-rGO_ae_ negative electrode

GO was firstly synthesized using a modified Hummers method previously reported by our group^14^,^26^,^59^,^60^,^61^,^62^. The N-rGO_ae_ was then synthesized via a hydrothermal reduction of GO with 0.5 M hydrazine (N_2_H_4_) a reducing agent. First, the as-synthesized GO (160 mg) was dispersed in Milli-Q water (80 ml) using a sonication process (100 w) for 2 h. N_2_H_4_ was then added to the mixture at room temperature. The mixture was consequently transferred to a Teflon autoclave (100 ml) and heated at 110 °C for 24 h to form N-rGO hydrogel. For the purification, the as-synthesized hydrogel was immersed in Milli-Q water to remove the residuals for 72 h. Finally, the hydrogel was frozen at 0 °C for 24 h. Then, the frozen hydrogel was put in a freezing dryer to remove water at −55 °C for 48 h. The product is so-called N-rGO_ae_. In order to fabricate the negative electrode, the as-synthesized N-rGO_ae_ (3 mg) was dispersed in ethanol (3 ml), spray-coated on the c-CFP using an airbrush with a 0.3-mm brush nozzle (Paasche Airbrush Company, USA) and eventually dried at 50 °C for 24 h.

### Potential-step electrodeposition of the MnO_2_ positive electrode

The c-CFP at a diameter of 1.58 cm was immersed in an electrodeposition solution, 30 ml of 250 mM Mn(NO_3_)_2_·H_2_O in 250 mM KCl. MnO_2_ nanosheets were electrodeposied on the c-CFP by a potential-step electrodeposition at 1.0 V vs. SCE for 3 min and then suddenly switched to 0.5 V vs. SCE for 1 min for which a chronoamporometry method was carried out using a potentiostat (PGSTAT 302 N). In order to have 1–2 mg of MnO_2_, this process was repeated for 10 times. Finally, the as-electrodeposited electrode was then washed 3 times with Milli-Q water to remove the residual KCl and dried at 50 °C for 24 h.

### Morphological and structural characterizations

X-ray diffraction (XRD) using a D8 ADVANCE with DAVINCI design (Bruker optics, Germany) with CuK_α_ of 1.5418 Å was used to characterize the crystalline structures of the as-synthesised materials i.e., GO, N-rGO_ae_, and MnO_2_. The data were collected from 5 to 80° (2θ) with 0.01 increment. Note, the Si wafer was used as a holder for XRD measurement. Raman spectroscopy was also carried out using a laser excitation wavelength of 532 nm (Senterra Dispersive Raman, Bruker optics, Germany). The field-emission scanning electron microscopy (FE-SEM) images of the as-prepared materials were performed with an accelerating voltage of 15.0 kV (JSM-7001F, JEOL Ltd., Japan). The samples were mounted on the clean surface of carbon conductive tab and placing on the SEM pin stub. Note that the specimens were coated with the platinum by a sputtering technique for 40 sec in order to remove the charging effect. The transmission electron microscopy (TEM) images of the samples were performed with an accelerating voltage of 100 kV (a JEM 1220, JEOL Ltd., Japan). The TEM specimens were prepared by dropping the suspension (∼0.05 mg/ml) of N-rGO_ae_, and MnO_2_ in ethanol in the copper grids and dried at 50 °C for 3 h. The functional groups and elemental compositions of the as-synthesised materials were also analyzed by X-ray photoelectron spectroscopy (XPS) using an AXIS Ultra DLD (Kratos Analytical Ltd., Manchester, UK) with Al-K alpha radiation (*hv* = 14,866 eV). In addition, *in situ* Mn K-edge fluorescent x-ray photoelectron spectroscopy (XAS) measurement was performed at a beamline No. 5 at the Synchrotron Light Research Institute (Public Organization), Nakhon Ratchasima, Thailand using a Ge(220) double-crystal monochromator (energy range 3440–12100 eV). The spectroscopic data were collected in fluorescence mode with a 4-element silicon drift detector. The 4-element silicon drift detector was placed 90° to the beam and 45° to the sample. The Mn K-edge (6539 eV) was calibrated using the Mn foil before measurement. The light dimension on the sample was adjusted to 5 mm width and 1 mm height. The advantage of using *in situ* XAS measurements is that it can probe or localize the Mn element of the MnO_2_ electrode during charging/discharging.

For the *in situ* electrochemical XAS measurement, a chronoamperometry method was used at different potentials (i.e., 0, 0.4, and 0.8 V vs. SCE) to evaluate the electrochemical property of the electrodes. In this measurement, a 3-electrode system using SCE as a reference electrode, Pt wire as a counter electrode, and the as-prepared MnO_2_ working electrode was carried out in a 0.5 M Na_2_SO_4_ (aq.) electrolyte. Note, the electrochemical cell was made from acrylic sheets with the dimension of 2 × 2 × 3.5 cm^3^ having a drilled hole diameter of 0.8 cm on one 2-cm^2^ side of the acrylic sheet. The drilled hole was covered by a larger piece of Kapton tape with a diameter of 1.2 cm. The SCE and Pt wire were placed beside the MnO_2_ electrode at a distance of ca.1 cm but away from the path of the X-rays. In order to get a steady-state current, the MnO_2_ working electrode was kept at a given potential of interest for at least 15 min before the *in situ* XAS and chronoamperometry measurements.

### Fabrication of ASCs and the electrochemical evaluation

The ASCs were assembled of the negative and positive electrodes with a coin-cell size (CR2016). Hydrolyzed polyethylene (PE) film with a thickness of 25 μm was used as the separator of aqueous-based supercapacitors and 0.5 M Na_2_SO_4_(aq) was used as the electrolyte. The electrolyte seperator was prepared by soaking the hydrolized PE in 0.5 M Na_2_SO_4_(aq) for 10 min before assembled. Then, the separator was inserted betweent positive and negative electrode. Finally, the coin cell was then assembled by pressing with crimper machine at 100 psi. The electrochemical evaluation of the as-fabricated supercapacitors was carried out using a Metrohm AUTOLAB potentiostat (PGSTAT 302 N) made in Netherlands running NOVA software (version 1.11). Cyclic voltammetry (CV), galvanostatic charge–discharge (GCD), and electrochemical impedance spectroscopy (EIS) were performed.

### Calculation of supercapacitor performances

The specific capacitance (C_cv_) of the supercapacitor cell excluding the influence of the c-CFP substrate can be determined from the CV by following calculation Eq. (8)^40^,^63^,^64^,^65^.


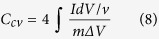


where *ΔV* is the working potential determined from the discharge potential chosen in the potential range without H_2_ and O_2_ evolution, *IdV* is an area under the discharging curve, 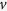 is a scan rate (V/s), and *m* is a total active mass at negative and positive electrodes (g).

The specific capacitance can also be calculated from the GCD method (*C*_*GCD*_) by following Eq. (9)^40^,^65^,^66^;


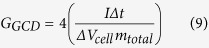


where *I* is the applied constant current (A), *Δt* is the discharging time (s), and Δ*V*_*cell*_ is the potential window (V) excluding *iR* drop. Note, the *iR* drop increases when increasing the applied current rate.

The specific capacitance determined from the EIS technique (*C*_*EIS*_) can be calculated from Eq. (10)^2^,^67^,^68^;


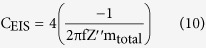


where *f* is the applied frequency and *Z*″ is the imaginary component of the impedance at the frequency *f*, which is a negative value. In addition, the equivalent series resistance (ESR) of the supercapacitors was simply determined from the intercept at the X-axis of the Nyquist plots.

Besides, the specific energy (*E*) of the supercapacitors were calculated by following Eq. (11)^69^,^70^,^71^;


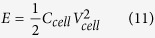


The maximum power (*P*_*max*_) at the discharge efficiency of 50% from a maximum voltage at the fully charged state can be calculated as the following Eq. (12)^69^ ;


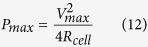


where *V*_*max*_ is a maximum voltage of the cell, and *R*_*cell*_ is a resistance of the cell, which can be determined from the *iR*_*cell*_ drop observed in the GCD^13^.

## Additional Information

**How to cite this article**: Iamprasertkun, P. *et al*. Charge storage mechanisms of manganese oxide nanosheets and N-doped reduced graphene oxide aerogel for high-performance asymmetric supercapacitors. *Sci. Rep*. **6**, 37560; doi: 10.1038/srep37560 (2016).

**Publisher’s note:** Springer Nature remains neutral with regard to jurisdictional claims in published maps and institutional affiliations.

## Supplementary Material

Supplementary Information
