# Supplementary material for: Charge storage mechanisms of manganese oxide nanosheets and N-doped reduced graphene oxide aerogel for high-performance asymmetric supercapacitors
Source: Sci Rep. 2016 Nov 18;6:37560. doi: 10.1038/srep37560 (PMC5114613; doi:10.1038/srep37560)
Supplement: Supplementary Information [file srep37560-s1.doc]

Supporting Information

Charge storage mechanisms of manganese oxide nanosheets and N-doped reduced graphene oxide aerogel for high-performance asymmetric supercapacitors

Pawin Iamprasertkun^1,2^, Atiweena Krittayavathananon^1^, Anusorn Seubsai^2^, Narong Chanlek^3^, Pinit Kidkhunthod^3^, Winyoo Sangthong^4^, Santi Maensiri^5^, Rattikorn Yimnirun^5^, Sukanya Nilmoung^6^, Panvika Pannopard^7^, Somlak Ittisanronnachai^7^, Kanokwan Kongpatpanich^8^, Jumras Limtrakul^8^ and Montree Sawangphruk^1,*^

^1^Department of Chemical and Biomolecular Engineering, School of Energy Science and Engineering, Vidyasirimedhi Institute of Science and Technology, Rayong 21210, Thailand
^2^Department of Chemical Engineering, Kasetsart University, Bangkok 10900, Thailand

^3^Synchrotron Light Research Institute (Public Organization), 111 University Avenue, Muang District, Nakhon Ratchasima 30000, Thailand

^4^Department of Chemistry, Kasetsart University, Bangkok 10900, Thailand

^5^School of Physics, Institute of Science, Suranaree University of Technology, Nakhon Ratchasima 30000, Thailand

^6^Department of Applied Physics, Faculty of Sciences and Liberal Arts, Rajamangala University of Technology Isan, Nakhon Ratchasima 30000, Thailand

^7^Frontier Research Centre (FRC), Vidyasirimedhi Institute of Science and Technology, Rayong 21210, Thailand

^8^Department of Materials Science and Engineering, Vidyasirimedhi Institute of Science and Technology, Rayong 21210, Thailand


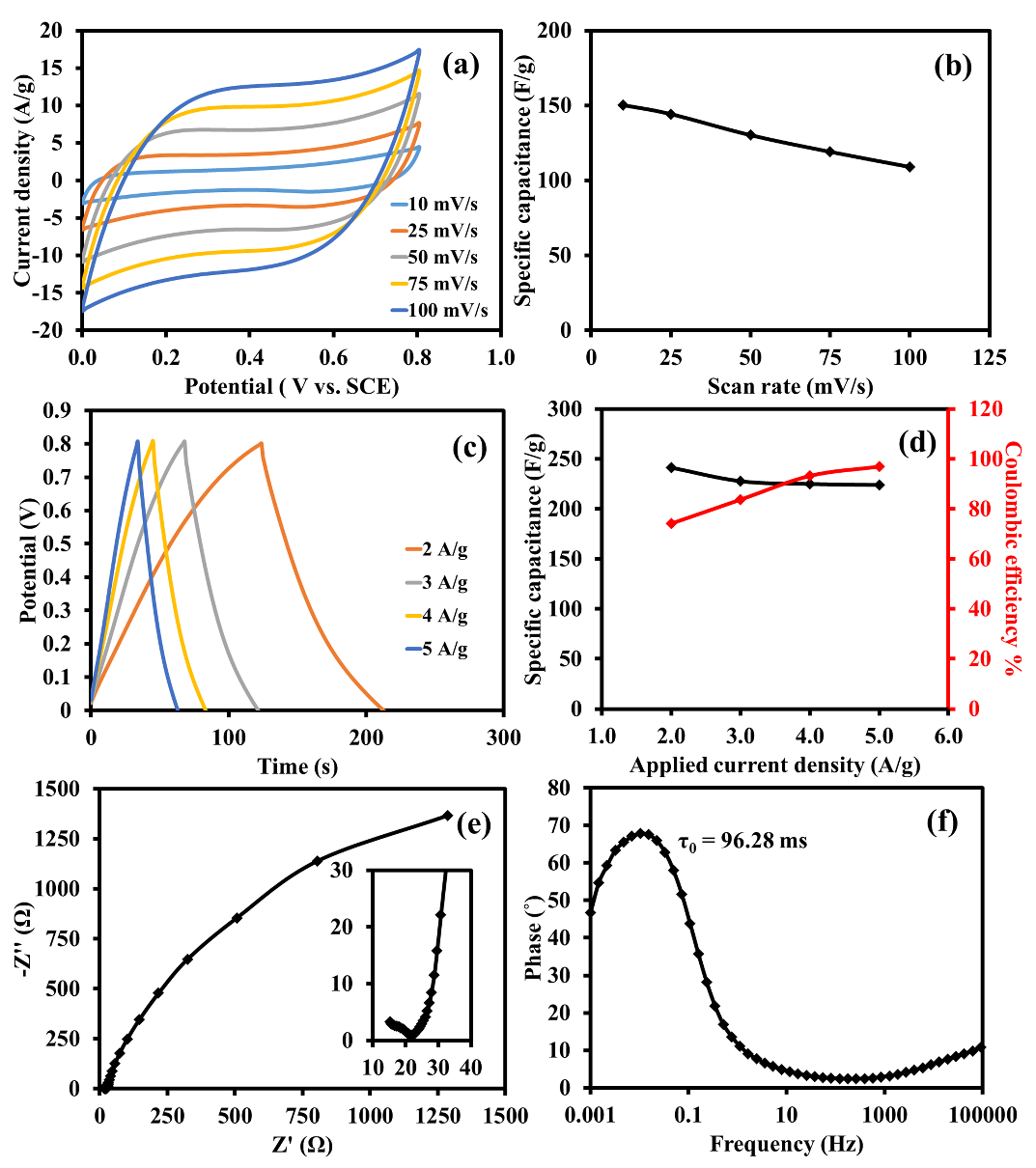


**Figure S1**. The electrochemical properties of the MnO_2_/c-CFP half-cell electrodes: (a) CVs, (b) specific capacitance vs. scan rates, (c) GCDs, (d) specific capacitance and coulombic efficiency vs. applied current density, (e) Nyquist plot, and (f) phase vs. frequency.


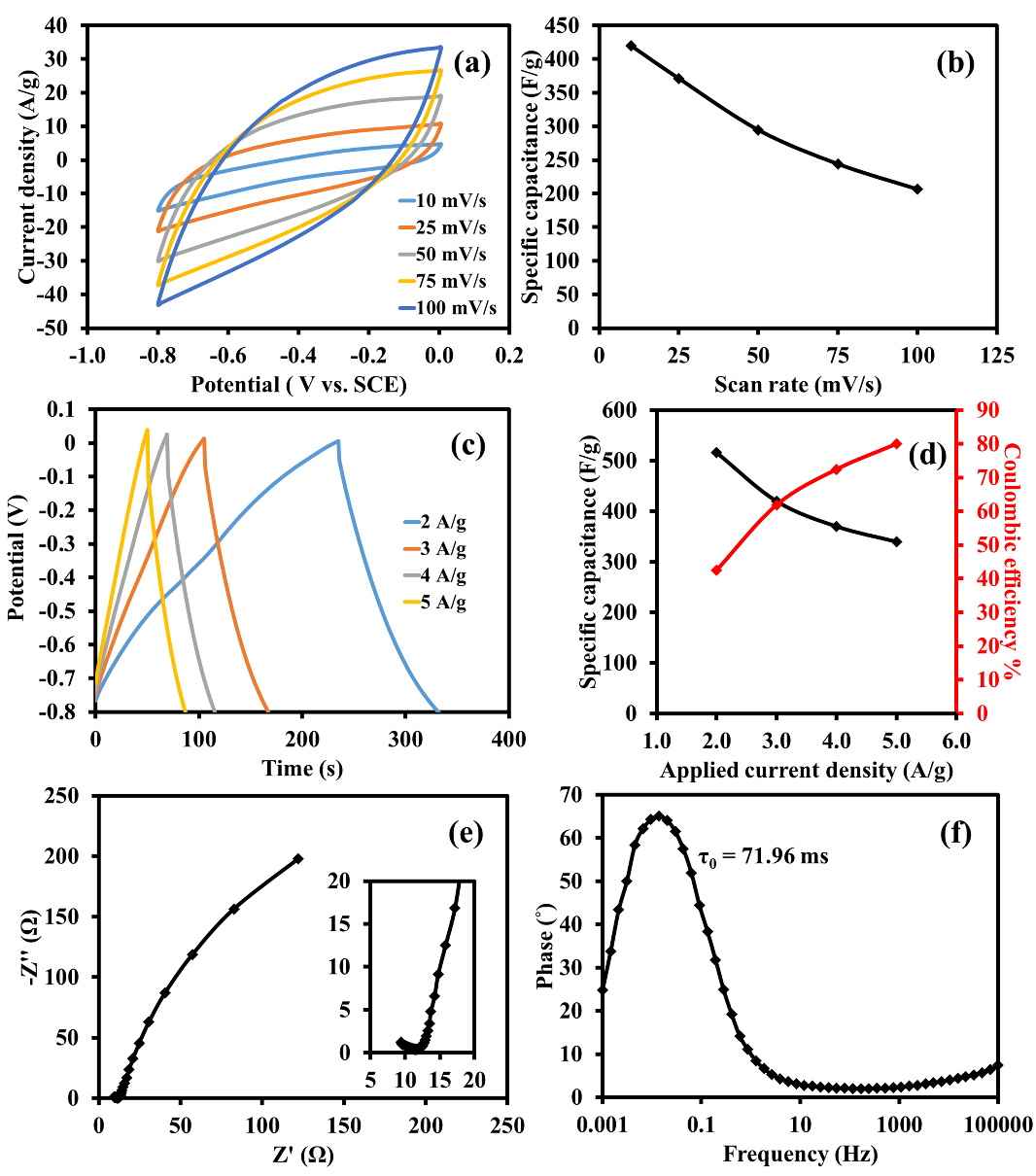


**Figure S2**. The electrochemical properties of N-rGO_ae_/c-CFP half-cell electrodes; (a) CVs, (b) specific capacitance vs. scan rates, (c) GCDs, (d) specific capacitance and coulombic efficiency vs. applied current density, (e) Nyquist plot, and (f) phase vs. frequency.


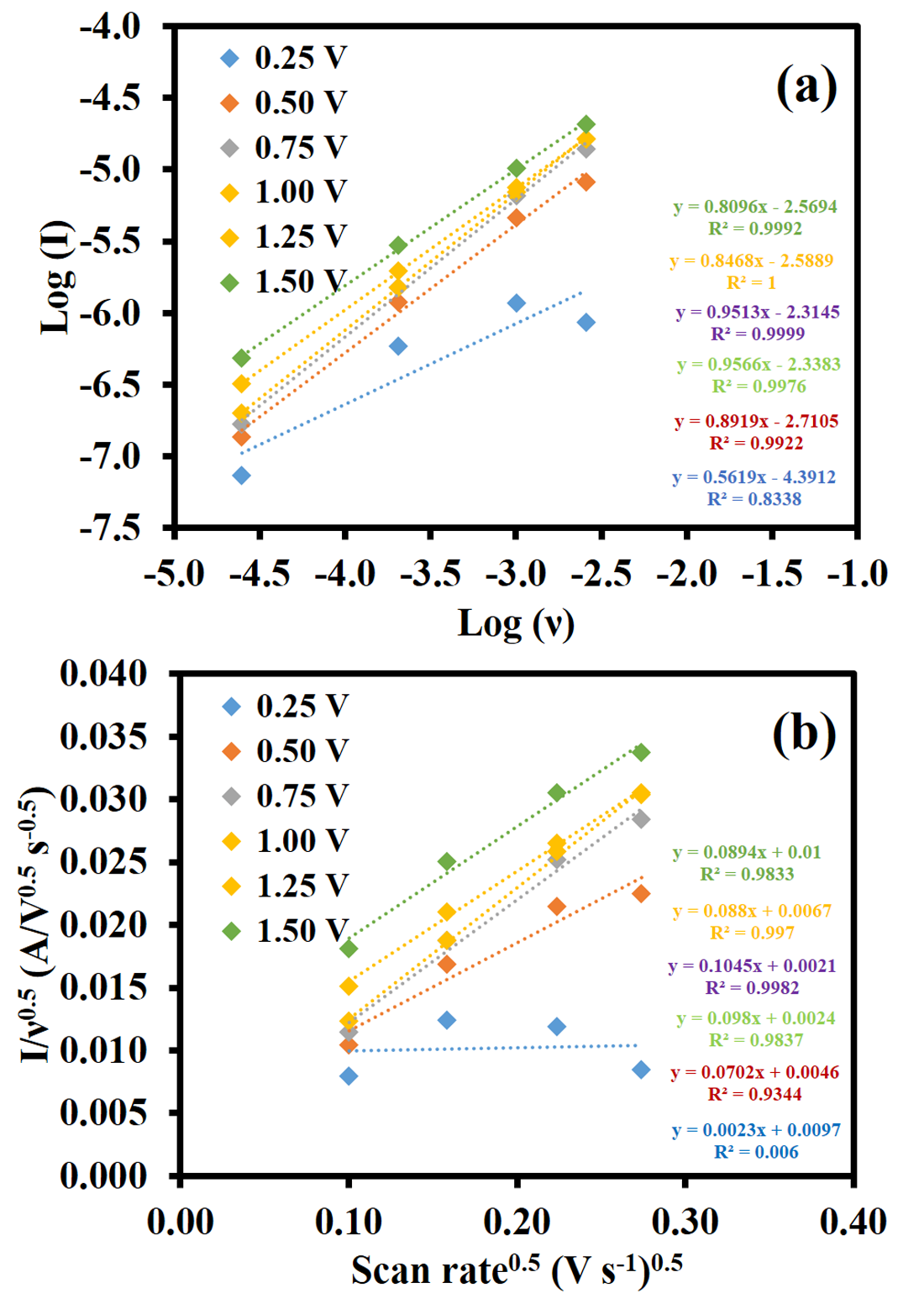


**Figure S3**. *(a)* log i*vs.* log ν and (b) I/ν ^0.5^ vs. ν^0.5^.


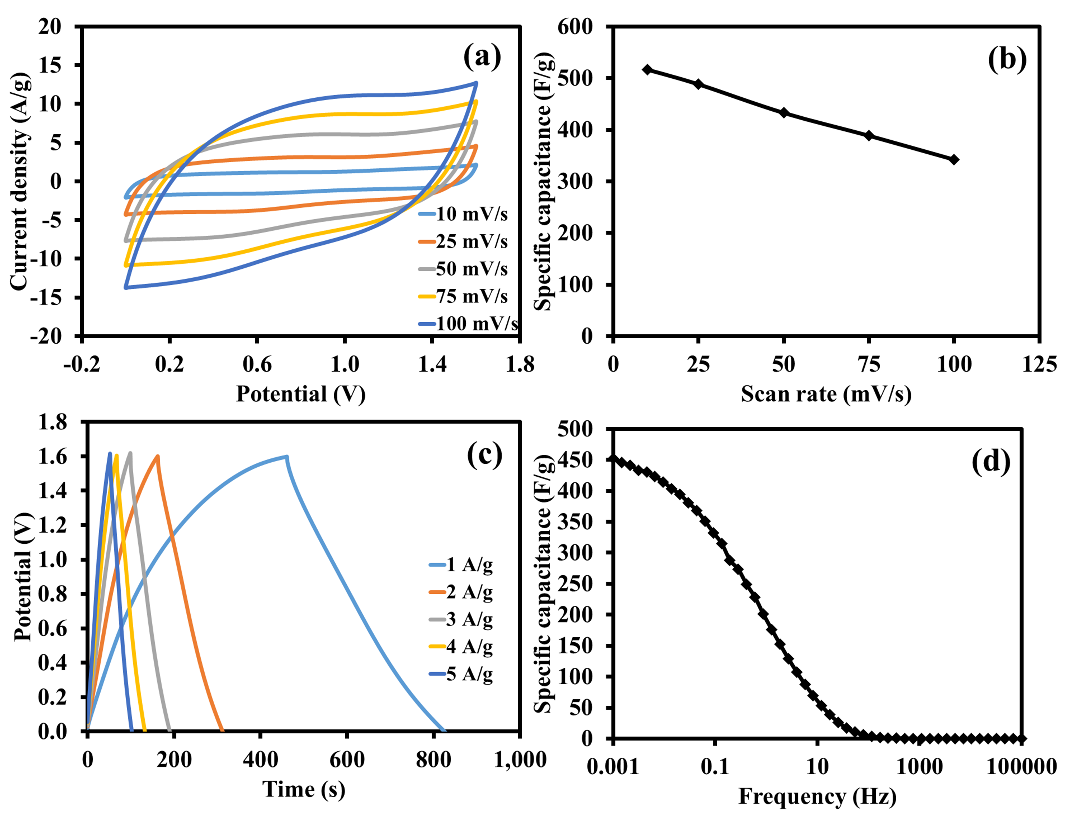


**Figure S4**. The electrochemical properties of the asymmetric supercapacitor cell; (a) CVs, (b) specific capacitance vs. scan rates, (c) GCDs, (d) specific capacitance vs. frequency.


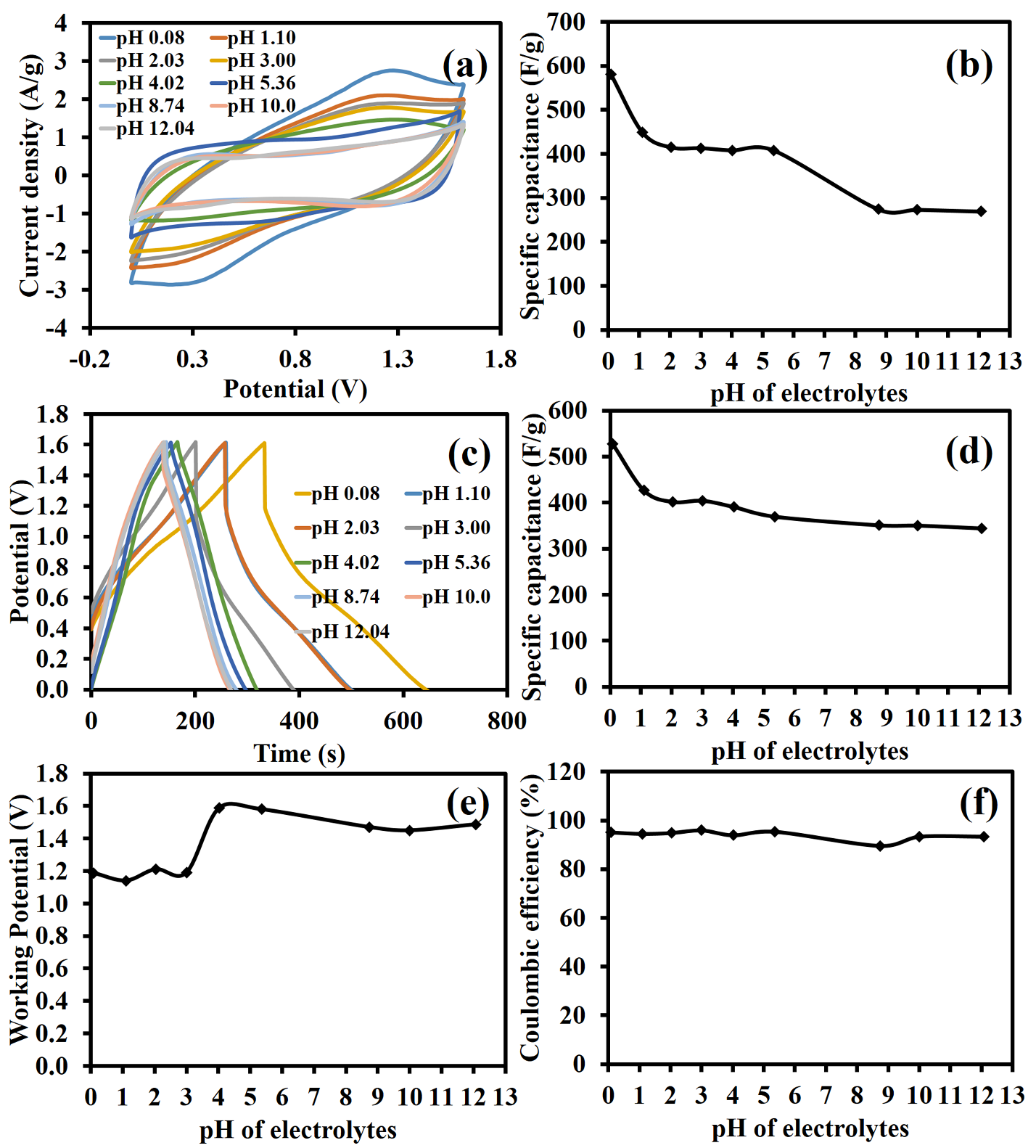


**Figure S5**. The electrochemical properties of the asymmetric supercapacitor cell at different pH; (a) CVs at 10 mV s^-1^, (b) specific capacitance (CV) vs. pH of electrolytes, (c) GCDs at 2 A g^-1^, (d) specific capacitance (GCD) vs. pH of electrolytes, (e) working potential vs. pH of electrolytes, and (f) Coulombic efficiency vs. pH of electrolytes at 2 A g^-1^.

**
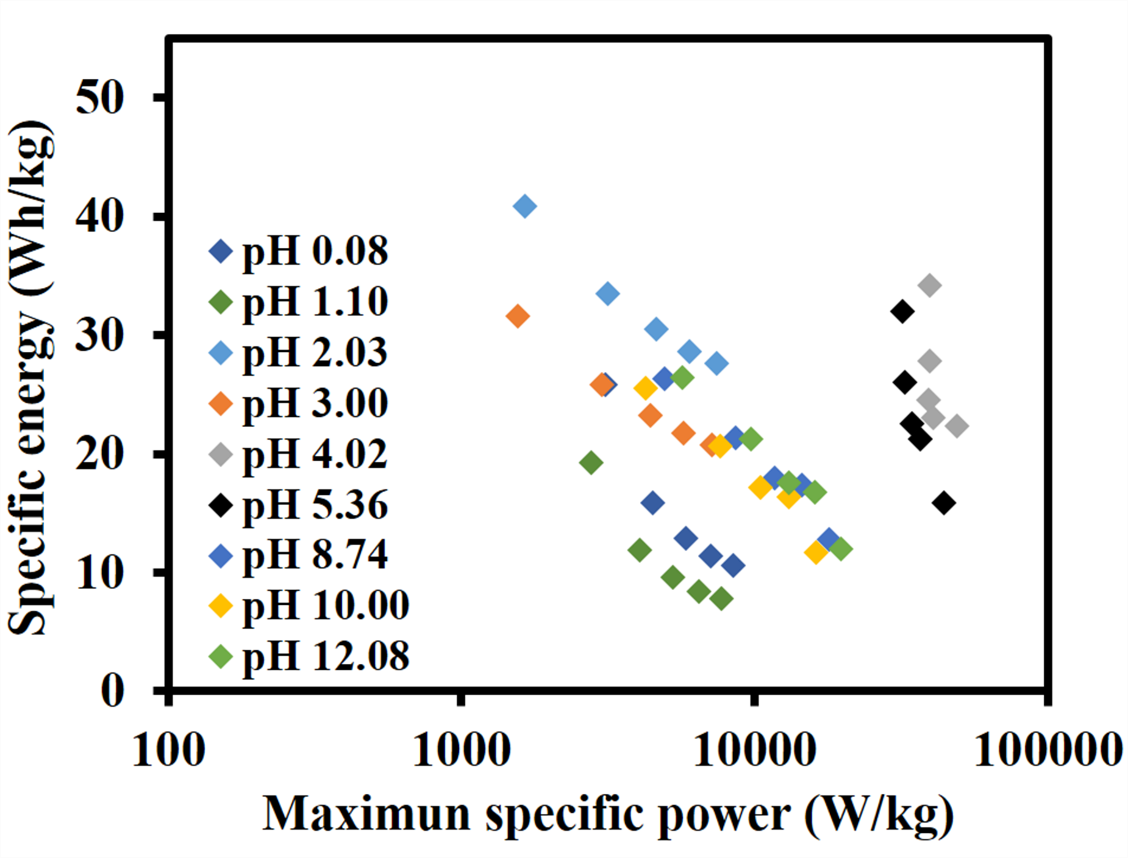
**

**Figure S6**. The ragone plot of the as-frabicated K-birnessite MnO_2_//N-rGO_ae_ cells at different pH 0.5 M Na_2_SO_4_ electrolytes.
